# Core promoter in TNBC is highly mutated with rich ethnic signature

**DOI:** 10.1093/bfgp/elac035

**Published:** 2022-10-28

**Authors:** Teng Huang, Jiaheng Li, Heng Zhao, Chumpol Ngamphiw, Sissades Tongsima, Piranit Kantaputra, Wiranpat Kittitharaphan, San Ming Wang

**Keywords:** core promoter, RNA-seq, triple-negative breast cancer, variation, exome sequences

## Abstract

The core promoter plays an essential role in regulating transcription initiation by controlling the interaction between transcriptional factors and sequence motifs in the core promoter. Although mutation in core promoter sequences is expected to cause abnormal gene expression leading to pathogenic consequences, limited supporting evidence showed the involvement of core promoter mutation in diseases. Our previous study showed that the core promoter is highly polymorphic in worldwide human ethnic populations in reflecting human history and adaptation. Our recent characterization of the core promoter in triple-negative breast cancer (TNBC), a subtype of breast cancer, in a Chinese TNBC cohort revealed the wide presence of core promoter mutation in TNBC. In the current study, we analyzed the core promoter in a Thai TNBC cohort. We also observed rich core promoter mutation in the Thai TNBC patients. We compared the core promoter mutations between Chinese and Thai TNBC cohorts. We observed substantial differences of core promoter mutation in TNBC between the two cohorts, as reflected by the mutation spectrum, mutation-effected gene and functional category, and altered gene expression. Our study confirmed that the core promoter in TNBC is highly mutable, and is highly ethnic-specific.

## Introduction

Gene expression is under tight control by the regulatory machinery consisting of transcriptional factors and DNA sequence motifs through their *cis*–*trans* interaction. Core promoter is the region located surrounding the transcription start site (TSS) and is essential for gene expression regulation through controlling transcriptional initiation. Eukaryotic core promoter is enriched with highly conserved motifs such as TFIIB recognition element (BRE), TATA box, initiator element (INR), downstream promoter element (DPE) and transcription factor binding sites (TFBS). The interaction between the DNA motifs (*cis*-) and transcriptional initiation complex of RNA polymerase II and other factors (*trans*-) in the core promoter controls transcription initiation [1–3]. Genetic variation in core promoter sequences can have a drastic impact on transcriptional initiation by altering the *cis*–*trans* interaction; pathogenic mutations in the core promoter region can contribute to pathogenic consequences including cancer [4–6].

While the core promoter has been extensively studied and its roles in gene expression regulation have been well established biologically, the importance of the core promoter in human diseases has not been well studied. Core promoter mutation in *TERT* in melanoma is one of the few examples of core promoter mutation and diseases. *TERT* codes for telomerase reverse transcriptase involved in telomere structure. Mutation in the *TERT* core promoter causes TERT overexpression in several types of cancer including melanoma and bladder cancer [7–9] (Most of the gene expression studies in diseases focused on measuring the changes at mRNA or protein levels.). The cause for the altered gene expression by the regulatory abnormality including core promoter mutation is largely ignored. This can be partly explained by the technical difficulty to study the core promoter due to its compact structure highly conserved between different genes [10]. To understand the potential biological roles of core promoter variation, we characterized the core promoter in worldwide human populations [11]. The results revealed a highly polymorphic nature of core promoter in the human population highlighting the importance of the core promoter variation in human evolution adaptation. To understand the potential pathogenic impact of the core promoter mutation, we characterized the core promoter mutations in breast cancer and bladder cancer [12, 13]. The results showed the wide presence of germline and somatic core promoter mutations in both breast cancer and bladder cancer, suggesting that core promoter mutations could play etiological roles in oncogenesis in different types of cancer.

The presence of both germline and somatic mutations in the core promoter raises an interesting question. That is whether the core promoter mutation in the same type of cancer could be universally present regardless of the ethnic background or ethnic-specific. The answer to this question is essential to understand the etiological roles of core promoter mutation in cancer and to provide a precise clinical diagnosis. Triple-negative breast cancer (TNBC) is a class of breast cancer. It is characterized by the absence of estrogen receptor (ER), progesterone receptor (PR) and human epidermal growth factor receptor 2 (HER2) [14–16]. TNBC has distinct features from other types of breast cancer by early onset, aggressive, metastatic and poor prognosis. Gene expression in TNBC is also very different from other types of breast cancer [17–19]. In our previous study, we systematically analyzed core promoter mutation in Chinese TNBC patients and identified germline and somatic mutations in thousands of genes [13]. In the current study, we analyzed TNBC patients from Thailand to address the question of whether core promoter mutation in the same type of cancer could be ethnic-specific or universally present regardless of the ethnic background. Genomic data clearly show the unique genetic features of the Thai population [20], and TNBC in the Thai population has its unique mutation signature [21]. Through mapping analysis using the genomic sequences from Thai TNBC patients and filtering the normal polymorphism of core promoter variation using core promoter variation data including Thai and other populations, we identified the core promoter mutations in Thai TNBC patients. Comparing the core promoter mutation data between Thai and Chinese Han TNBC patients, we observed the commonly shared but also substantially different core promoter mutations between the two TNBC cohorts. Our study revealed that the core promoter is highly mutated in TNBC but the mutation signature is diversified in different ethnic populations.

## Material and methods

### Data sources

Whole exome sequencing data (*n* = 116) were from Thai TNBC patients [21] (https://www.ncbi.nlm.nih.gov/sra, SRP178744). RNA-seq data (*n* = 360) and paired normal tissues (*n* = 88) were from the TNBC study [16] (https://www.ncbi.nlm.nih.gov/sra, SRP157974). Human genome reference sequences hg19 were used as the references for core promoter mapping analysis.

### Identification of core promoter variants

The EVDC method was used to collect the core promoter sequences from exome sequences as described in detail in reference [22]. Briefly, reference core promoter sequences and coordinates from hg19 were extracted by using BEDTools utility (version 2.27.1) [23]. Core promoter sequences from control and TNBC samples were extracted from the corresponding exome sequence data with the following steps: the exome sequences were converted to FASTQ format by using the SRA Toolkit (version 2.9.1) [24]; BWA utility (version 0.7.17) [25] was used to map exome sequences to the human genome reference sequences (hg19); the resulting SAM files were converted into BAM files and sorted using SAMtools (version 1.9) [26]; duplicates were removed and read group information was added using Picard (version 2.18.25) [27]; the BAM files were further processed using Genome Analysis Toolkit (version 4.1.1.0) [27] for variant calling; the called variant files were compressed and indexed by using the BCFtools utility (version 1.9) [26] and annotated using ANNOVAR Toolkit [28] for gene-based and filter-based annotations. The filter-based annotation was used to determine known or novel variants and allele frequencies. Variants matched in databases of dbSNP, 1000 Genome, ESP, ExAC, gnomAD and ClinVar were considered germline variants. The polymorphic germline variants were filtered using the core promoter variant data derived from Thai population [20] and multiple populations including the 1000 Genome Project East Asian (EAS) [8] and other resources [8, 20, 29–35] (Supplementary Table 1). The variants present in >58 cases (50%, somatic) and 6 cases (5%, germline) of the 116 TNBC cases were classified as normal polymorphic variants and eliminated; the variants present only in single cases were considered as private variants and eliminated. The remaining variants were considered TNBC-derived mutations and used for downstream analysis.

### Gene Ontology analysis

For the genes with core promoter mutation, their sequences, functional categories and pathways were analyzed using Gene Ontology (GO) knowledgebase [36], GeneCards [37] and National Center for Biotechnology Information database [38]. GO terms were identified using Metascape [39]. Stemness feature analysis on pan-cancer was performed by using the Sangerbox platform (http://vip.sangerbox.com/). The TCGA Pan-Cancer [40] data set (*n* = 10 535) were derived from the UCSC [41] database. The prognostic summary in cancer tissues was searched in Human Protein Atlas [42].

### Differential gene expression analysis

RNA-seq data from TNBC and paired normal samples were used for the analysis. Differential gene expression between cancer and normal samples was determined by using HISAT2 (version 2.2.0) [43], SAMtools (version 1.9) [26], StringTie (version 2.1.1) [44] and DESeq2 (version 1.26.0) [45] following the instructions in each program. Volcano plots were used to show differential expressed genes using R ggplot2 package [46].

### Statistics analysis

Student’s *t*-test was used to compare the mutation types between cancer and non-cancer samples, with a *P*-value <0.05 as significant difference. Benjamini–Hochberg adjusted *P*-value <0.05 and fold changes ≥1.5 were considered significant differences for the differentially expressed genes. *P*-value <0.05 by using the hypergeometric test and overlap ≥3 were considered significant in enrichment analysis. Similarity score >0.3 was used to link the terms in the GO cluster network. Pearson correlation was used in the stemness feature analysis, log2 (*x* + 0.001) transformation was performed on each expressed value and *P*-value <0.05 was considered significant. Venn comparisons between populations were generated using Venny [47] (https://bioinfogp.cnb.csic.es/tools/venny/index.html).

## Results

### Core promoter variation in Thai TNBC patients

We collected the core promoter sequences from 116 Thai TNBC patients [21]. We called variants in the collected core promoter sequences. After extensive filtering with the core promoter variants derived from Thai and non-Thai populations, we removed the polymorphic variants from the called core promoter variants and identified somatic and germline core promoter mutations. In total, we identified 9232 recurrent somatic mutations (present in ≥2 carriers, 80 mutations per TNBC case on average) composed of 932 distinct somatic variants in 619 core promoters of 649 genes (Table 1A and Supplementary Table 2). The most frequent mutation type was substitution (56.7%) and 99.0% were absent in the COSMIC database. We also identified 734 recurrent germline mutations (present in ≥2 carriers, 6 mutations per TNBC case on average), composed of 220 distinct mutations in 108 core promoters of 119 genes (Table 1A and Supplementary Table 3), with the most frequent type of insertion (38.6%). There was no sharing of the same position between somatic and germline mutations. However, 21 variant-containing genes shared both somatic and germline mutation groups. The total number of genes with somatic and germline core promoter mutation was 746, around 3.7% of the total genes in the human genome.

**Table 1:** Summary of variants identified in core promoters

Items	Core promoter variants	
	Somatic	Germline
A. General features		
Total	9232	734
Distinct	932	220
Co-promoter with variants	619	108
Absent in COSMIC database	923	220
Type		
Substitution	528	51
Insertion	181	85
Deletion	223	84
Gene affected	649	119
Average number of mutation/case	80	6
B. Variation frequency in motifs		
Total	1161	1455
MTE_box2	400	493
DCE_box3	142	91
BREd	124	146
DPE	102	77
Inr	96	55
TCT	63	219
Ets	49	38
DTIE	45	60
MTE_box1	36	9
BREu	27	23
E-Box	24	10
DCE_box2	20	18
DCE_box1	19	209
SP1	7	0
XCPE1	6	5
TATA box	1	2
TCF	0	0
XCPE2	0	0
C. Transition, transversion and Ts/Tv ratio		
Transition		
G>A	88	15
C>T	78	7
A>G	17	4
T>C	9	2
Total	192	28
Transversion		
C>A	93	7
G>C	21	4
C>G	21	1
A>C	13	2
G>T	63	2
T>G	5	3
A>T	13	1
T>A	11	3
Total	240	23
Ts/Tv ratio^a^	1.60	2.43

^a^Ts/Tv ratio was calculated by 2xTs/Tv.

The core promoter mutations were highly enriched at core promoter motifs (Table 1B). For example, there were 400 somatic mutations and 493 germline mutations located at the MTE box2 motif. Consistent with its highly stable nature [22], only one somatic and two germline mutations were located in the TATA box. For the 114 single-base germline substitutions, the transition (Ts)/ transversion (Tv) ratio was 2.43 (Table 1C), significantly lower than the 3.25–3.81 in CHB (Han Chinese in Beijing), 3.65; in CHS (Southern Han Chinese), 3.28; in CDX (Chinese Dai in Xishuangbanna), 3.25; in JPT (Japanese in Tokyo), 3.81; in KHV (Kinh in Ho Chi Minh City), 3.37 in EAS (East Asian) populations from the 1000 Genome data [11]. We compared the distribution of mutations in motif and non-motif regions in the core promoter. The results showed significant differences between two regions (somatic mutation: *p*-value = 0.01167 and germline mutation: *p*-value = 0.01536 by Student’s *t*-test), indicating that the mutations in the motifs were not randomly distributed.

**Figure 1:**
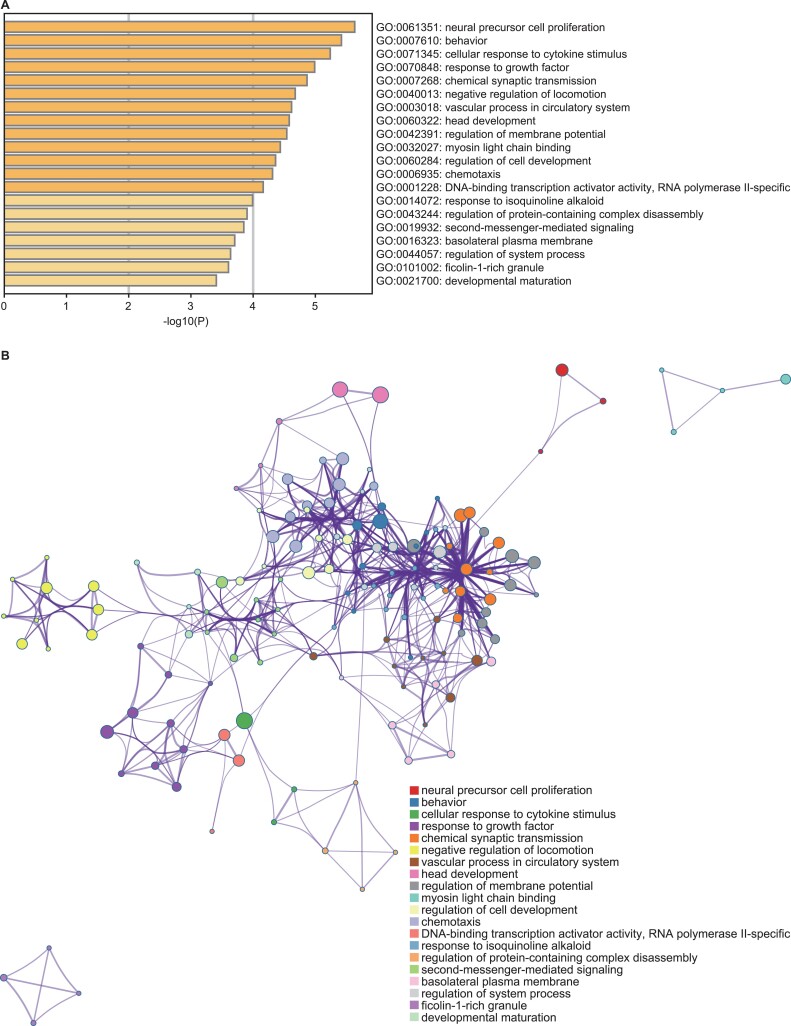
GO classification of core promoter mutated genes with altered expression. (**A**) Example of GO classification; (**B**) cluster network of representative terms of GO classification. Circle nodes represent terms, and their colors represent their clusters. Terms with a similarity score >0.3 are linked by edges.

We performed functional annotation for the core promoter mutated genes and observed that the affected genes were enriched with multiple functional pathways relevant to cancer development, such as ‘Response to tumor necrosis factor’, ‘Cell population proliferation’, ‘Negative regulation of cell migration’, ‘Positive regulation of reproductive process’, ‘Response to growth factor’, ‘Inflammatory response’, ‘Regulation of vasoconstriction’, ‘Regulation of cell differentiation’ and ‘Chemotaxis’ (Figure 1 and Supplementary Table 4).

### Effects of core promoter mutation on gene expression

**Figure 2:**
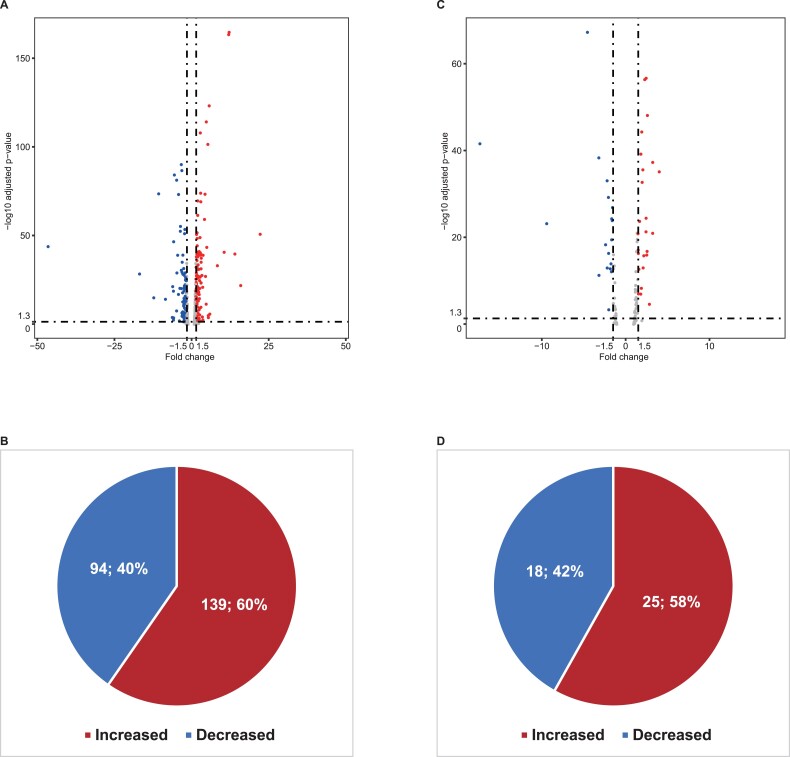
Core promoter mutated genes with altered gene expression in TNBC. The volcano plot showed the differential expression of genes with core promoter mutation in TNBC and non-TNBC based on RNA-seq data. *X*-axis represents fold changes of increased or decreased expression, and *Y*-axis represents the distribution of the genes with altered expression at −log10 scale (adjusted *P*-value). Red dots: Increasingly expressed genes with statistically significant. Blue dots: Decreasingly expressed genes with statistically significant. The pie chart displays the number of differential expression genes. (**A**) Differential expression of the genes with somatic core promoter mutation. (**B**) Proportion of genes of somatic core promoter mutation with altered gene expression. (**C**) Differential expression of genes with germline core promoter mutation. (**D**) Proportion of genes of germline core promoter mutation with altered gene expression.

To test if core promoter mutation can alter gene expression, we compared the expression of the genes in TNBC to the non-cancer control and searched the differential expression of genes with core promoter mutations. Of the 932 genes with somatic core promoter mutations in TNBC, 233 (25.0%) had altered expression including 139 increased and 94 decreased expression, with *IGF2BP1* as the highest of 22.4-fold increased expression and *MYOC* as the highest of 46.4-fold decreased expression (Figure 2A and B, and Supplementary Table 5); of the 220 genes with germline core promoter mutation in TNBC, 43 (19.5%) had altered expression including 25 with increased and 18 with decreased expression, with *SLC11A1* as the highest of 4.1-fold increased expression and *TRHDE-AS1* as the highest of 17.3-fold decreased expression (Figure 2C and D, and Supplementary Table 6). We compared the expression between the genes without core promoter mutation and with core promoter mutation. The results showed the presence of statistically significant differences (with core promoter-somatic mutation: *P*-value <2.2e−16 by Fisher’s exact test) and germline mutation (with core promoter-germline mutation: Fisher’s exact test: *P*-value <2.2e−16 by Fisher’s exact test).

**Table 2:** Examples of functional important genes with core promoter mutation

Gene	Position	#Carrier	Fold change	Breast cancer gene panel	Type
A. Examples of cancer-related genes					
*IGF2BP1*	−40	5	+22.4		Somatic
*HMGA1*	−34	2	+5.4		Somatic
*EIF5A*	−96	2	+2.1		Somatic
*MSN*	−24	2	+1.6		Somatic
*FTH1*	+28	2	+1.6		Germline
*ING1*	−92	34	+1.5		Somatic
*ARHGEF2*	+55	2	+1.5		Somatic
*ACVR2A*	+8	4	−1.7		Somatic
*TBX3*	+90	45	−2.0		Somatic
*MITF*	−49	2	−2.0		Germline
*PID1*	−57	2	−3.1		Germline
*HAS1*	+83	5	−3.1		Germline
*SLIT2*	+92	13	−3.5		Somatic
*ESR1*	−41	2	−4.6		Somatic
*KLF4*	+81	2	−4.7		Somatic
B. Signature genes included in breast cancer gene panels					
*S100P*	−66	2	+10.7	Sorlie500	Somatic
*MFAP2*	−77	17	+4.5	Hu306	Somatic
*MBOAT2*	−40	2	+2.1	Sorlie500	Somatic
*FAM110A*	−53	2	+2.0	Sorlie500	Somatic
*DCK*	+89	39	+1.8	MammaPrint	Somatic
*GPI*	−95	2	+1.8	Sorlie500	Somatic
*RAB3A*	−99	2	+1.8	Hu306	Somatic
*GALNT3*	+76	3	+1.7	Sorlie500	Somatic
*FZD6*	−45	26	+1.5	Sorlie500	Somatic
*CITED2*	−69	2	−1.7	Sorlie500	Somatic
*GRIA2*	+71	5	−2.0	Sorlie500	Germline
*LARP6*	+20	2	−2.2	Sorlie500	Somatic
*RGS5*	−1	2	−2.7	Sorlie500	Somatic
*ESR1*	−41	2	−4.6	PAM50|Sorlie500	Somatic

### Core promoter mutation in cancer-related genes

**Figure 3:**
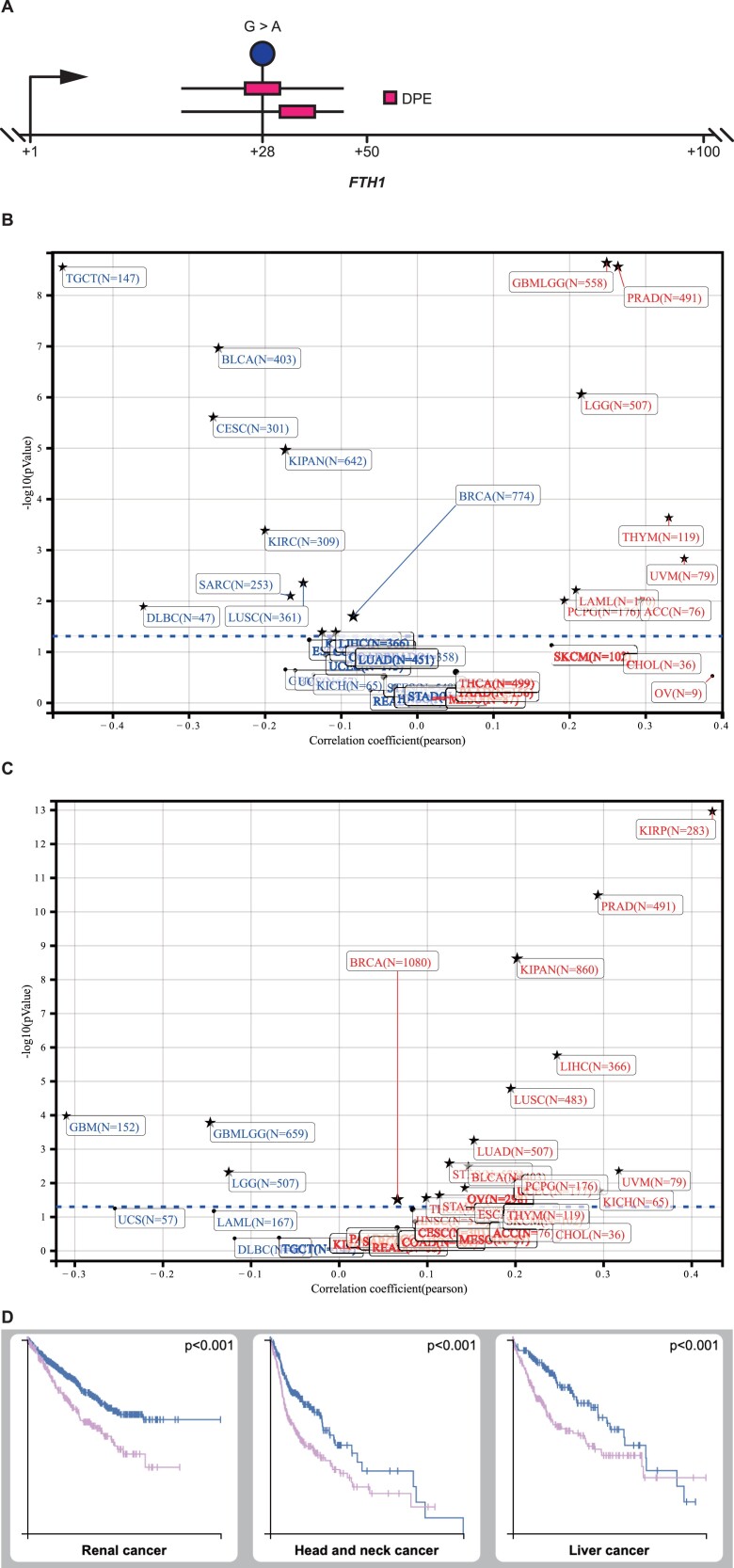
Core promoter mutation in *FTH1*. BRCA, breast cancer. Stars with red cancer names represent positive correlation and stars with blue cancer names represent negative correlation. (**A**) Core promoter mutation in *FTH1* in TNBC. The lollipop represents the mutation. The upper line represents the mutation-altered sequences, and the bottom line refers to the reference sequences. The boxes in the lines represent the mutated core promoter motifs. A GC>AC variant at +28 bp shifted a DPE motif from +30 to +26 bp in the core promoter of *FTH1*. (**B**) Negative correlation between epigenetically regulated, DNA methylation-based stemness and gene expression of *FTH1* in pan-cancer. (**C**) Positive correlation between epigenetically regulated, RNA expression-based stemness and gene expression of *FTH1* in pan-cancer. (**D**) Kaplan–Meier plots for high expression of *FTH1* showed significant association with patient survival in renal cancer, head and neck cancer and liver cancer. Pink line represents high expression and blue line represents low expression.

Of the genes with core promoter mutation, many are classical oncogenes or tumor suppressors, and associated with breast cancer (Table 2, and Supplementary Tables 2 and 3). For example, *ESR1* encodes an ER and ligand-activated transcription factor, which plays a key role in breast cancer, endometrial cancer and osteoporosis [48]. A G>A somatic mutation at 41 bp upstream of TSS (−41 bp) in two TNBC cases deleted a BREd in the *ESR1* core promoter, leading to a 4.6-fold decreased expression in TNBC over the control. IGF2BP1 is a member of the insulin-like growth factor 2 mRNA-binding protein family. It stabilizes CD44 mRNA through binding to the CD44 mRNA 3′-UTR and promotes cell adhesion and invadopodia formation in cancer cells [49]. A poly(T) simple repetitive sequence was deleted at its −40 bp in five TNBC cases, causing a 22.4-fold increased expression. GABRA3 is associated with non-small-cell lung cancer [50]. A ‘CTCTCTCTCTCTCT’-like simple repetitive sequence was inserted at its 30 bp downstream of the TSS (+30 bp) in four TNBC cases, causing a 16.1-fold increased expression. FTH1 is the heavy subunit of ferritin, which is the major intracellular iron storage and detoxifying protein [51]. A G>A germline variation in *FTH1* at +28 bp was present in two Thai TNBC carriers. This variant putatively shifted a DPE motif from +30 to +26 bp (Figure 3A) and modified the sequence of an Inr motif, leading to a 1.6-fold increased expression in TNBC (Supplementary Table 6). *FTH1* plays a role in the negative regulation of cell population proliferation and tertiary granule lumen (Supplementary Table 4). Stemness features have been found to be associated with oncogenic dedifferentiation [52]. We performed the stemness feature analysis on pan-cancer and found that *FTH1* expression was associated with the stemness features in many types of cancer including breast cancer. Epigenetically regulated, DNA methylation-based stemness (Figure 3B) was negatively correlated (Pearson correlation: *P*-value = 2.01e−02) with the gene expression of *FTH1* in breast cancer (*n* = 774). Epigenetically regulated RNA expression-based stemness (Figure 3C) was positively correlated (Pearson correlation: *P*-value = 2.97e−02) with the gene expression of *FTH1* in breast cancer (*n* = 1080). Additionally, FTH1 is an unfavorable prognostic marker in renal cancer, head and neck cancer and liver cancer (Figure 3D).

**Figure 4:**
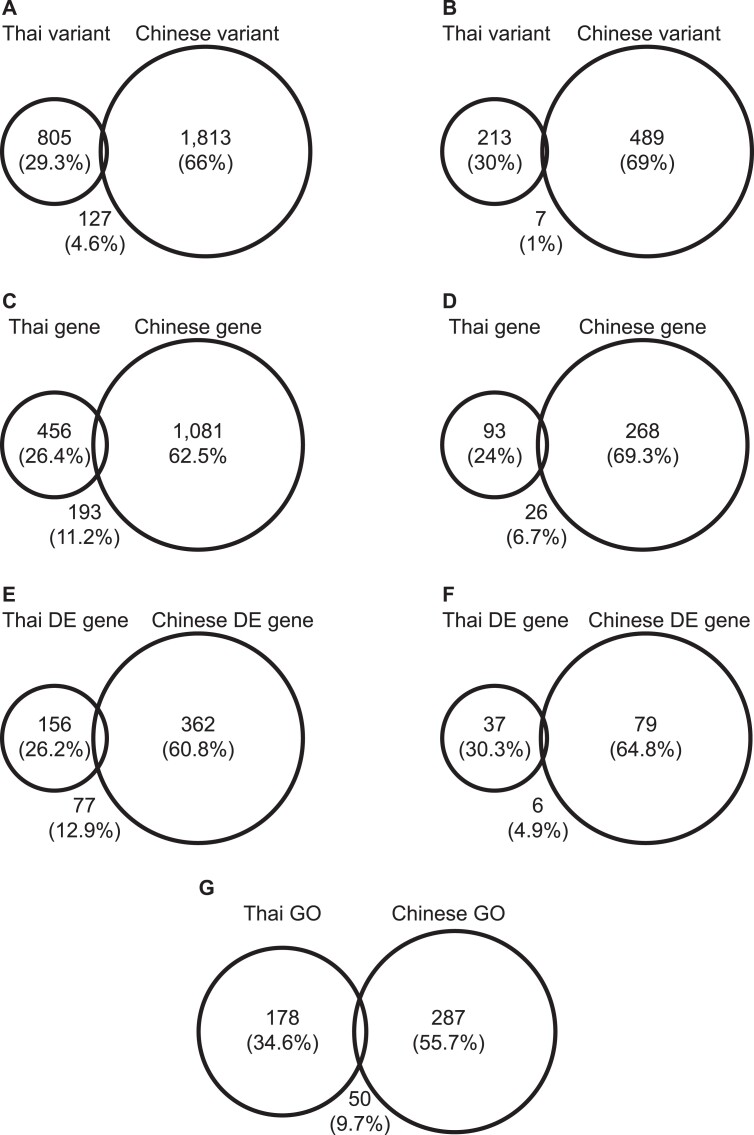
Comparison of core promoter mutation between Thai TNBC and Chinese TNBC cohorts. (**A**) Somatic core promoter mutation; (**B**) germline core promoter mutation; (**C**) somatic mutated genes; (**D**) germline mutated genes; (**E**) somatic mutation altered gene expression; (**F**) germline mutation altered gene expression. DE, differential expression. (**G**) GO classification for core promoter mutated genes.

### Altered gene expression in core promoter mutated genes

Gene expression in TNBC has been extensively studied by using gene panels specifically designed for breast cancer [53, 54], such as the Mamma Print [55], PAM50 [56], Hu306 [57] and Sorlie500 [58]. We searched the core promoter mutated genes in these panels and identified 14 core promoter-mutated genes with altered expression (Table 2B, and Supplementary Tables 5 and 6), indicating that core promoter mutation in TNBC can also contribute to altered gene expression in other types of breast cancer.

### Ethnical specificity of core promoter mutation in TNBC

We compared the core promoter mutation data between Thai TNBC (*n* = 116) and Chinese TNBC (*n* = 279) (Figure 4). The results showed substantial differences between the two data sets as reflected by

(i) Different mutation spectrum. Only 127 somatic mutations (13.6% of Thai and 6.5% of Chinese mutations) and 7 germline mutations (3.2% of Thai and 1.4% of Chinese mutations) were shared between the two cohorts (Figure 4A and B).
(ii) Different mutation-effected genes. Only 193 somatic mutated genes (29.7% of Thai and 15.1% of Chinese mutated genes) and 26 germline mutated genes (21.8% of Thai and 8.8% of Chinese) were shared between the two cohorts (Figure 4C and D).
(iii) Different altered expression of core promoter mutated genes. Altered expressed genes were shared only in 77 somatic mutated genes (33.0% of Thai and 17.5% of Chinese) and 6 germline mutated genes (14.0% of Thai and 7.1% of Chinese) between the two cohorts (Figure 4E and F).
(iv) Different functional categories of core promoter mutated genes. Only 50 GO-classified genes (21.9% of Thai and 14.8% of Chinese) were shared between the two cohorts (Figure 4G). Examples included ‘Response to growth factor’, ‘Positive regulation of cell development’ and ‘Cell maturation’.

Function enrichment analysis was performed by using Thai-specific mutated genes with altered expression. These functions were significantly associated with Thai-specific mutated genes, including ‘regulation of inflammatory response’, ‘response to tumor necrosis factor’, ‘negative regulation of cell motility’ and ‘cell population proliferation’. For example, ‘regulation of inflammatory response’ was present in six Thai-specific genes including *ESR1*; ‘Response to tumor necrosis factor’ was present in *EIF5A*, *GBA*, *RORA*, *CCL25* and *ARHGEF2* with somatic mutations; ‘Negative regulation of cell motility’ was present in *HAS1*, *MITF*, *LRCH1* with germline mutations. On the other hand, certain mutations were found in the mutated genes in Chinese TNBC. For example, somatic mutations were found in the core promoters of *BRCA2* and *FANCB* in Chinese TNBC but not in Thai TNBC (Supplementary Table 7A and B).

## Discussion

In this study, we performed extensive characterization of core promoter mutation in the TNBC from the Thai cohort. We then compared the core promoter mutation data with those from the Chinese TNBC cohort. The data from our study demonstrate that core promoter mutation of both somatic and germline is commonly present in TNBC affecting around 10% of the genes in the TNBC genome. The data from our study also reveal that the core promoter mutation in TNBC is highly ethnic-specific.

TNBC is a common type of breast cancer. Data from extensive genomic study in TNBC showed that the genomic abnormality in TNBC is highly diverse between the patients with different ethnic backgrounds, reflecting the influences of different genetic backgrounds in TNBC etiology [18, 19, 21, 59, 60]. Our study in the Chinese TNBC cohort revealed that core promoter mutation is commonly present in TNBC [13]. It is necessary to determine whether the core promoter mutation in TNBC follows the pattern of genetic mutation in another part of the TNBC genome, such as the coding region, or is more universally present in TNBC but not affected by ethnic background. Our current study addressed this issue by comparing the core promoter mutations between Thai and Chinese cohorts. The genetic background between the Thai and Chinese populations is substantially different. The Thai population is highly diversified in reflecting its genetic admixture with its neighboring population [61], whereas the Chinese population is relatively homogeneous in reflecting its relative stable evolutionary path in the East Asia region although having extensive internal heterogeneity [62, 63]. The same analytic conditions and extensive filtering of normal genetic polymorphism used in the two studies provided a quality control to ensure the reliability of the mutation data derived for the comparison between the two TNBC cohorts.

The core promoter is essential in gene expression regulation through controlling transcriptional initiation. Although the importance of core promoter has been well determined biologically, the rich knowledge has not been adequately extended to benefit medicine, as reflected by the lack of knowledge of core promoter mutation in oncogenesis. To investigate the potential roles of the core promoter in diseases, we first developed the EVDC method for studying core promoter sequences carried by exome sequence data [22]. We then investigated the core promoter polymorphism in 25 human ethnic populations using the exome data generated by the 1000 Genome project. We observed the highly diversified core promoter architecture in humans in reflecting its evolution and environmental adaptation [11]. Using the normal polymorphism data as the control, we further investigated the core promoter mutation in the Chinese TNBC cohort and the Spanish bladder cancer cohort. We observed that the core promoter region is highly mutable in cancer in a cancer type-specific manner [13]. In the current study, we further investigated the issue of ethnic background for core promoter mutation in the same type of cancer, TNBC. By using the core promoter mutation data from the Chinese and Thai cohort, our study further confirmed the wide presence of core promoter mutation in TNBC and observed the significant impact of ethnic genetic background on core promoter mutation. The information provides valuable insight for the etiology of oncogenesis in different ethnic populations and potential targets for clinical applications. Further study in more cancer types in different ethnic background will help to illustrate further the importance of core promoter mutation in cancer.

**Key Points**

- The core promoter in triple-negative breast cancer (TNBC) can be systematically characterized by using bioinformatics methods in multiple TNBC cohorts.
- The core promoter in TNBC is highly mutable in East Asians.
- The mutation signature is highly ethnic-specific as reflected by the mutation spectrum, mutation-effected gene and functional category, and altered gene expression.

## Ethics approval and consent to participate

Not applicable.

## Consent for publication

Not applicable.

## Data Availability

All data generated or analyzed during this study are included in this published article and its supplementary information files.

## Code availability

Public websites and code packages used in this study were described in the methods.

## Authors’ contributions

T.H.: Data curation, methodology, software, formal analysis, writing—original draft and visualization. J.L.: Formal analysis, investigation and writing—original draft. H.Z.: Data curation. C.N., S.T., P.K. and W.K.: Resources. S.M.W.: Conceptualization, methodology, writing—revision and funding acquisition.

## Supplementary Material

Supplementary_Table_1_elac035

Supplementary_Table_2_elac035

Supplementary_Table_3_elac035

Supplementary_Table_4_elac035

Supplementary_Table_5_elac035

Supplementary_Table_6_elac035

Supplementary_Table_7_elac035

## Author Biographies

**Teng Huang** is a PhD fellow in the Faculty of Health Sciences, University of Macau, Macau, China. His research interests focus on bioinformatics and gene expression regulation.

**Jiaheng Li** is a PhD student in the Faculty of Health Sciences, University of Macau, Macau, China. His research interests focus on evolution of DNA damage repair genes.

**Heng Zhao** is a master student in Data Sciences Program. He is working on genetic variation in *BRCA*.

**Chumpol Ngamphiw** is a researcher at the National Biobank of Thailand, National Science and Technology Development Agency, Thailand. His work focuses on bioinformatics, genome databases and computational genomics.

**Sissades Tongsima** is a principal researcher at the National Biobank of Thailand, National Science and Technology Development Agency, Thailand. His work focuses on bioinformatics, computational and population genomics.

**Piranit Nik Kantaputra** is a professor in the Center of Excellence in Medical Genetics Research, Faculty of Dentistry, Chiang Mai University, Thailand. His interest includes rare human malformations in dentition.

**Wiranpat Kittitharaphan** is a clinical doctor in Yuwapasartwaithayopathum Hospital, Samutprakarn, Thailand. Her interest is in pediatric psychiatry.

**San Ming Wang** is a professor in the Faculty of Health Sciences, University of Macau, Macau, China. His research focuses on germline variation in DNA damage repair genes and early prevention of hereditary cancer.
